# Serum Lipidomics Profiling to Identify Biomarkers for Non-Small Cell Lung Cancer

**DOI:** 10.1155/2018/5276240

**Published:** 2018-08-07

**Authors:** Yingrong Chen, Zhihong Ma, Xiongrong Shen, Liqin Li, Jing Zhong, Li Shan Min, Limin Xu, Hongwei Li, Jianbin Zhang, Licheng Dai

**Affiliations:** ^1^Huzhou Key Laboratory of Molecular Medicine, Huzhou Central Hospital, Huzhou, Zhejiang 313000, China; ^2^Departments of Clinical Pharmacology, Huzhou Central Hospital, Huzhou, Zhejiang 313000, China; ^3^Cardiothoracic Surgery, Huzhou Central Hospital, Huzhou, Zhejiang 313000, China

## Abstract

Non-small cell lung cancer (NSCLC) is the leading cause of cancer death worldwide, which ranks top in both incidence and mortality. To broaden our understanding of the lipid metabolic alterations in NSCLC and to identify potential biomarkers for early diagnosis, we performed nontargeted lipidomics analysis in serum from 66 early-stage NSCLC, 40 lung benign disease patients (LBD), and 40 healthy controls (HC) using Ultrahigh Performance Liquid Chromatography-Quadrupole Time-of-Flight Mass Spectrometry (UHPLC-Q-TOF/MS). The identified biomarker candidates of phosphatidylcholines (PCs) and phosphatidylethanolamines (PEs) were further externally validated in a cohort including 30 early-stage NSCLC, 30 LBD, and 30 HC by a targeted lipidomic analysis. We observed a significantly altered lipid metabolic profile in early-stage NSCLC and identified panels of PCs and PEs to distinguish NSCLC patients and HC. The levels of PCs and PEs were found to be dysregulated in glycerophospholipid metabolism, which was the top altered pathway in early-stage NSCLC. Receiver operating characteristic (ROC) curve analysis revealed that panels of PCs and PEs exhibited good performance in differentiating early-stage NSCLC and HC. The levels of PE(16:0/16:1), PE(16:0/18:3), PE(16:0/18:2), PE(18:0/16:0), PE(17:0/18:2), PE(18:0/17:1), PE(17:0/18:1), PE(20:5/16:0), PE(18:0/18:1), PE(18:1/20:4), PE(18:0/20:3), PC(15:0/18:1), PC(16:1/20:5), and PC(18:0/20:1) in early-stage NSCLC were significantly increased compared with HC (p<0.05). Overall, our study has thus highlighted the power of using comprehensive lipidomic approaches to identify biomarkers and underlying mechanisms in NSCLC.

## 1. Introduction

Lung cancer is the leading cause of cancer death worldwide, which ranks top in both incidence and mortality [1]. Non-small cell lung cancer (NSCLC), which accounts for 80% of all lung cancer cases, involves adenocarcinoma (ADC), squamous cell carcinoma (SqCC), and large cell carcinoma. Despite the great progress made against NSCLC in recent years, the five-year survival rate of NSCLC is 15% approximately [2, 3]. Currently, NSCLC clinical diagnosis mainly depends on chest X-rays and computed tomography, but these techniques have low sensitivity and specificity. Biopsy is not desirable to frequently detect tumor because of its invasiveness [4–6]. In addition, the common tumor biomarkers used in NSCLC, such as carcinoembryonic antigen (CEA) and cytokeratin 19 fragment (CYFRA21-1), show poor diagnostic values, which are not suitable for early detection of NSCLC [7–9]. Therefore, it is necessary to search for novel biomarkers for the early diagnosis of NSCLC.

Metabolomics can adapt nonconventional technology to tumor biomarker research and has been used in pharmacological analysis and disease diagnosis [10]. As an important branch of metabolomics, lipidomics is a system-based study of all lipids aiming at comprehensive analysis of lipids in the biological system [11–13]. Lipids are the fundamental components of biological membranes as well as the metabolites of organisms, which play a critical role in cellular energy storage, structure, and signaling [14–16]. The lipid imbalance is closely associated with numerous human lifestyle-related diseases, such as atherosclerosis [17], obesity [18], diabetes [19], Alzheimer's disease [20], and cancer [21]. Lipidomics has been accepted as a lipid-related research tool in lipid biochemistry [22], clinical biomarker discovery [21], and disease diagnosis [23] and in understanding disease pathology [24]. Lipidomics will not only provide insights into the specific functions of lipid species in health and disease, but will also identify potential biomarkers for establishing preventive or therapeutic programs for human diseases. The application of lipidomics in NSCLC biomarker discovery provides the opportunity for gaining novel insights into biochemical mechanism of NSCLC [25]. It has been reported that phospholipid and sphingolipid profiles changed in NSCLC, which may have important biological implications and may have significant potential for biomarker development [26–28]. But, up to now, few researches have clarified the changes of lipid profiles among early-stage NSCLC, lung benign disease, and healthy controls, and the potential lipid biomarkers for early diagnosis have also not been found. HPLC-MS has been widely used in lipidomics because it provides accurate qualitative and quantitative analysis. In this study, we used UHPLC-Q-TOF/MS to profile, identify, characterize, and quantify lipid compounds because of its high scanning speed, resolution, and sensitivity.

To broaden our understanding of the metabolic alterations, especially the lipid metabolic alterations in NSCLC and to identify potential biomarkers for early diagnosis, untargeted lipidomics evaluation was performed in sera from 66 early-stage NSCLC (35 ADC and 31 SqCC), 40 LBD, and 40 healthy controls (HC). In the subsequent pathway analysis, glycerophospholipid (GPL) pathway emerged at the top of these significantly altered metabolic pathways. The identified biomarker candidates of phosphatidylcholines (PCs) and phosphatidylethanolamines (PEs) were further externally validated in a cohort including 30 early-stage NSCLC, 30 LBD, and 30 HC by a targeted lipidomic analysis.

## 2. Materials and Methods

### 2.1. Chemicals

Liquid chromatography grade acetonitrile, methanol (MeOH), MTBE, and dichloromethane were purchased from Merck (Darmstadt, Germany). Ultrapure water was prepared by Milli-Q system (Millipore; Billerica, MA, USA). Lipidomix Mass Spec Standard (Catalog no. 330707, containing 160 *μ*g/mL phosphatidylcholine (15:0/18:1) (d7) and 5 *μ*g/mL phosphatidylethanolamine (15:0/18:1) (d7)) was purchased from Avanti Polar lipids (Alabaster, AL, USA).

### 2.2. Patients and Sample Collection

Serum samples were collected from NSCLC, LBD patients, and HC at Huzhou Central Hospital from January 2015 to July 2016. The patients were selected according to the following criteria: (1) all patients were diagnosed and confirmed by pathology; (2) patients with NSCLC were at the early stages (Stages I, II) according the clinical staging method; (3) patients had no other diseases which might affect lipid metabolism such as hyperlipidemia, diabetes, and other cancers; and (4) none of the patients received preoperative adjuvant chemotherapy or radiotherapy. LBD are defined as benign nodules, epithelioid granuloma, hamartoma, and inflammatory lesions. Serum samples from HC were collected from healthy volunteers with no history of carcinoma. Histopathology results for all cancer patients were confirmed by surgical resection of the tumors, while clinicohistopathological characteristics and tumor stages were assessed based on biopsy results. No preoperative chemotherapy or radiotherapy was administered to the cancer patients included in this study.

All samples were collected in accordance with ethical guidelines, and written informed consent was received. All patients were approached based on approved ethical guidelines, and patients who agreed to participate in this study were required to sign consent forms before being included in the study. The study was approved by Research Ethics Committee of Huzhou Center Hospital (No. 20150801). We also confirmed that all methods were performed in accordance with the relevant guidelines and regulations.

Before the collection of serum samples, patients and healthy volunteers fasted at least 12 hours. Briefly, for serum isolation, blood was collected into “increased silica act clot activator, silicone-coated interior, BD Vacutainer” and centrifuged at 700 g for 10 min at 4°C within 2 hours of venipuncture. The supernatant was removed and centrifuged in the same way for the second time. The resultant serum was transferred into a clean tube and stored at -80°C until use.

### 2.3. Nontargeted Lipidomics

#### 2.3.1. Sample Preparation

To perform the serum lipid analysis, 100 *μ*L of sample was added to 480 *μ*L of extraction liquid (V_MTBE_: V_methanol_ = 5:1) and vortexed for 30 s. The mixtures were allowed to stand for 20 min and then centrifuged at 3000 rpm for 15 min. A 400 *μ*L of the supernatant (MTBE extract) was transferred to a clean vial and dried in a vacuum concentrator. Dried samples were reconstituted with 100 *μ*L of dichloromethane/methanol (1:1, v/v).

#### 2.3.2. Chromatography and Mass Spectrometry

Lipid profiling was performed by a UHPLC system (1290 series, Agilent Technologies, USA) coupled with a quadruple time-of-flight mass spectrometer (Triple TOF 6600, AB SCIEX, USA). Phenomenex Kinetex C18 100 A column (1.7 *μ*m, 2.1×100 mm) (Phenomenex, USA) was used for the lipid extracts separation. The column was maintained at 25°C. The linear gradient started from 60% to 0% A (10 mmol/L ammonium formate, ACN: H2O = 6:4) and 40% B (10 mmol/L ammonium formate, IPA: H_2_O = 9:1). Gradient conditions were as follows: 0–12 min linear gradient from 40 to 100 % B, 12–13.5 min 100 % B. The flow rate was 300 *μ*L/min. The injected sample volume was 1 *μ*L. Data acquisition and processing were performed with the acquisition software Analyst TF (version 1.7.1, AB SCIEX, USA), which could acquire high resolution MS and tandem-MS data simultaneously by TOF MS full scan and information-dependent acquisition (IDA) in both ESI(+) and ESI(−) modes. The source parameters were set as follows: GAS1: 60 psi; GAS2: 60 psi; CUR: 30 psi; TEM: 250°C; ISVF: 5500 V in positive mode and -4500 V in negative mode, respectively, DP: 100 V, CE: 10 eV. MS raw data files were converted into the mzXML format using MSconverter, and processed by R package XCMS (version 1.41.0). The preprocessing results generated a data matrix that consisted of the retention time (RT), mass-to-charge ratio (m/z), and peak intensity. R package CAMERA was used for peak annotation after XCMS data processing [29]. Lipids identification was made by matching the acquired MS/MS data against MS/MS data in in-house developed database. The cutoff for match score was set as 0.8 and the minfrac was set as 0.5. All the m/z errors are less than 30 ppm and all the RT errors are less than 60 s. The data were normalised and the distribution was evaluated by MetaboAnalystR.

#### 2.3.3. Statistical Analyses

Data were presented as mean ± SD. SIMCA-P 14.1 (Umetrics, Umca, Sweden) was employed for multivariable analysis, including the principal components analysis (PCA) with mean-centered (ctr) scaling and orthogonal partial least squares discriminant analysis (OPLS-DA) with unit variance (uv) scaling. PCA was first used to reduce the dimensionality of the multidimensional dataset, while giving a comprehensive view of the clustering trend for the multidimensional data. OPLS-DA was then used to understand global lipid changes among NSCLC, LBD patients, and HC, and corresponding variable importance in the projection (VIP values) was calculated in OPLS-DA model as well. A sevenfold cross-validation method was used based on the OPLS-DA model to estimate the robustness and the predictive ability of our model. Potential metabolic biomarkers were selected with a VIP value greater than 1, and a* p* value of Student's* t*-test less than 0.05. In addition, the differentially abundant metabolites were cross-referenced to the pathways by further searching commercial databases, including KEGG (http://www.genome.jp/kegg/) and MetaboAnalyst (http://www.metaboanalyst.ca/).

#### 2.3.4. Selection of Metabolites for Targeted Lipidomics

Many factors were considered to select the appropriate lipid metabolites for targeted lipidomics. Because metabolomics is the study of metabolic profiles in living systems, the affected metabolic pathways containing affected metabolites were the principal criteria for selecting the biomarkers. In addition, the similarity values for the accuracy of compound identification and the number of differentially abundant metabolites detected in each test sample were important reference factors.

### 2.4. Targeted Lipidomics

#### 2.4.1. Sample Preparation

40 *μ*L of each sample was added to 160 *μ*L of water and 480 *μ*L of extraction liquid (V_MTBE_: V_methanol_ = 5:1, containing 10 *μ*L of 160 *μ*g/mL PC(15:0/18:1)) and vortexed vigorously for 60 s. The mixtures were ultrasound treated for 10 min and centrifuged at 3000 rpm for 15 min at 4°C. A 200 *μ*L aliquot of the supernatant was taken. To the lower liquid 200 *μ*L of MTBE was added and it was vortexed vigorously for 60 s. The mixtures were ultrasound treated for 15 min centrifuged at 3000 rpm for 15 min at 4°C. A 200 *μ*L aliquot of the supernatant was taken twice. The three supernatants (MTBE extract) were transferred to a clean vial and dried in a vacuum concentrator. Dried samples were reconstituted with 80 *μ*L of dichloromethane/methanol (1:1, v/v) and subjected to UHPLC-MS/MS analysis. 6 *μ*L of each sample was taken and pooled as quality control (QC) samples.

#### 2.4.2. Chromatography and Mass Spectrometry

Lipid profiling was performed by a UHPLC system (1290 series, Agilent Technologies, USA) coupled with a quadruple time-of-flight mass spectrometer (Triple TOF 6600, AB SCIEX, USA). Phenomenex Kinetex C18 100 A column (1.7 *μ*m, 2.1×100 mm) (Phenomenex, USA) was used for the lipid extracts separation. The column was maintained at 25°C. The linear gradient started from 60% to 0% A (10 mmol/L ammonium formate, ACN: H2O = 6:4) and 40% B (10 mmol/L ammonium formate, IPA: H_2_O = 9:1). Gradient conditions were as follows: 0–12 min linear gradient from 40 to 100 % B, 12–13.5 min 100 % B. The flow rate was 300 *μ*L/min. The injected sample volume was 1 *μ*L. Data acquisition and processing were performed with the acquisition software Analyst TF (version 1.7.1, AB SCIEX, USA), which could acquire high resolution MS and tandem-MS data simultaneously by TOF MS full scan and information-dependent acquisition (IDA) in both ESI(+) and ESI(−) modes. The source parameters were set as follows: GAS1: 60 psi; GAS2: 60 psi; CUR: 30 psi; TEM: 600°C; -4500 V in negative mode; CE: 45± 25 eV.

#### 2.4.3. Data Processing

The data was processed by an absolute quantitative lipidomics method [30]. MS raw data files were converted to the mzXML format using MSconverter and processed by R package XCMS (version 1.41.0). The preprocessing results generated a data matrix that consisted of the retention time (RT), mass-to-charge ratio (m/z), and peak intensity. Lipids identification was made by matching the acquired MS/MS data against MS/MS data in in-house developed database. The cutoff for match score was set as 0.8 and the minfrac was set as 0.5. All the m/z errors are less than 30 ppm and all the RT errors are less than 60 s. The metabolic features detected less than 50 % in all the QC samples were discarded [31]. The absolute concentrations (ng/ml) of each PC and PE were calculated based on the peak areas of the PC and PE identified in the sample and the peak areas of the internal standards of PC(15:0/18:1) and PE(15:0/18:1) corresponding to the sample.

#### 2.4.4. Statistics Analysis

SPSS version 19.0 (SPSS Inc., Armonk, NY, USA) was used for statistical analyses. Data were presented as mean ± SD. The differences on the levels of PC and PE among the three groups were evaluated by one-way analysis of variance (ANOVA) with Fisher's least significant test. ROC curve analysis was used to calculate the area under the ROC curve (AUC), sensitivities, and specificities. Differences were considered statistically significant when* p* values were less than 0.05 and fold change was larger than 1.5.

## 3. Results

### 3.1. Clinical Characteristics of the Study Samples

A total of 66 NSCLC patients including 35 ADC and 31 SqCC (mean age 61.5±8.2 years), 40 LBD (mean age 59.2±10.0 years), and 40 sex- and age-matched HC (mean age 54.0 ± 7.3 years) were included in our nontargeted lipidomics study. For the targeted lipidomics study, 30 NSCLC patients, 30 LBD, and 30 HC were included and there was no diversity, such as age and gender between the two groups. The clinical characteristics are summarized in Table 1.

### 3.2. Nontargeted Lipidomics Analysis

#### 3.2.1. Lipid Profiles of Serum Samples from Healthy Controls, Lung Benign Disease Patients, and Early-Stage NSCLC

**Figure 1:**
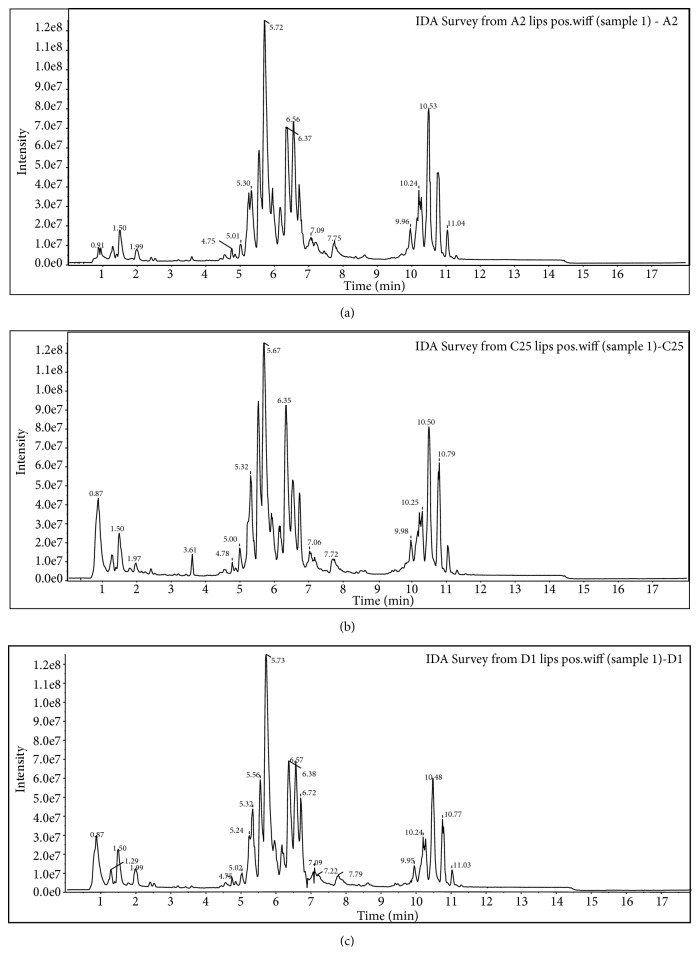
Typical TICs of lipid profiles of early-stage NSCLC, LBD, and HC by UHPLC-Q-TOF/MS analysis. ((a) NSCLC; (b) LBD; (c) HC). NSCLC, non-small cell lung cancer; LBD, lung benign disease; HC, healthy controls.

**Figure 2:**
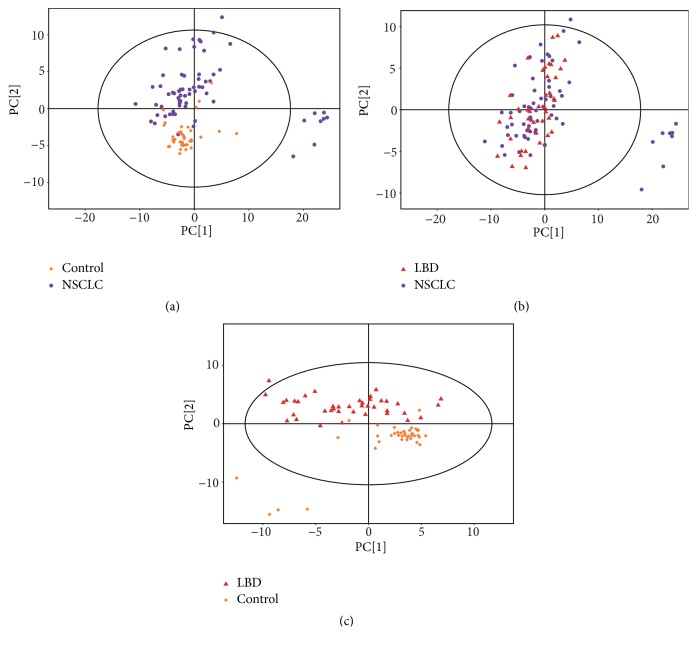
Principal component analysis (PCA) score plot of metabolic profile of early-stage NSCLC, LBD, and HC after mean-centering and not (Ctr) scaling ((a): NSCLC versus HC, R^2^X=0.560; (b) NSCLC versus LBD, R^2^X=0.531; (c) LBD versus HC, R^2^X=0.514). Purple dot, orange diamond, and red triangle denote early-stage NSCLC, HC, and LBD samples, respectively. NSCLC, non-small cell lung cancer; LBD, lung benign disease; HC, healthy controls.

**Figure 3:**
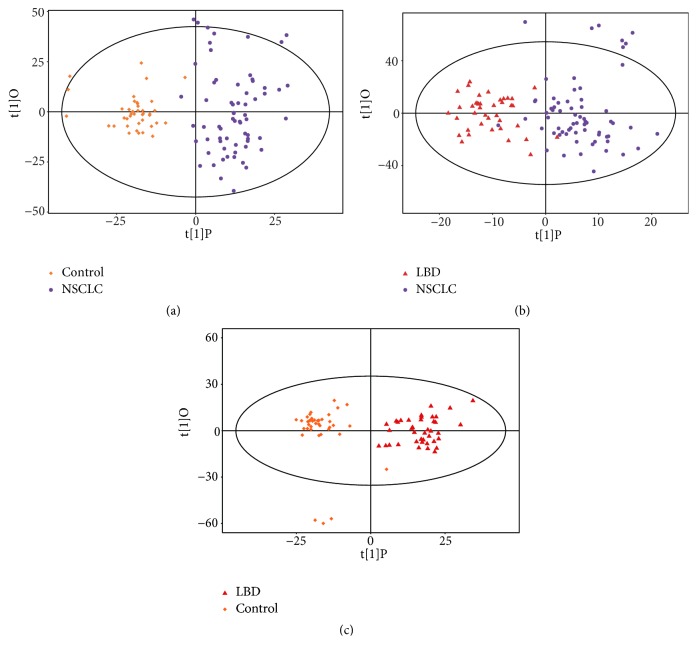
OPLS-DA score plot of lipid profile of early-stage NSCLC, LBD, and HC after unit variance (uv) scaling ((a) NSCLC versus HC; (b) NSCLC versus LBD; (c) LBD versus HC). NSCLC, non-small cell lung cancer; LBD, lung benign disease; HC, healthy controls.

Serum lipid profiles, including 493 lipid species in positive ion mode and 324 lipid species in negative ion mode were selected by nontargeted lipidomics from a total of 146 serum samples (66 NSCLC, 40 LBD, and 40 HC). There were 15 cholesteryl ester (CE), 16 ceramide (Cer), 7 diacylglycerol (DG), 10 dihexosylceramide (Hex2Cer), 7 hexosylceramide (HexCer), 1 2-monoglyceride (MG), 16 phosphatidic acid (PA), 214 PCs, 14 PCs with alkyl substituent (PC-O), 61 PCs with alkenyl substituent (PC-P), 72 PEs, 21 PE with alkenyl substituent (PE-P), 22 phosphatidylglycerol (PG), 45 phosphatidylinositol (PI), 43 sphingomyelin (SM), 1 sphingosine (SP), and 129 triglyceride (TG). Typical total ion chromatography (TICs) of lipid profiles is provided in Figure 1. The PCA score plots obtained for NSCLC group, LBD group, and HC group are shown in Figure 2. PCA revealed a clear separation between NSCLC patients and HC (Figure 2(a)). The parameters of the OPLS-DA score plots (Figure 3) were showed in Table 2. As shown in Figure 3(a), the OPLS-DA score plot revealed a clear separation between NSCLC patients and HC, with good fitting and predictive performances (R^2^Y = 0.803, Q^2^Y =0.739).

#### 3.2.2. Discovery and Identification of Potential Lipid Biomarkers

**Figure 4:**
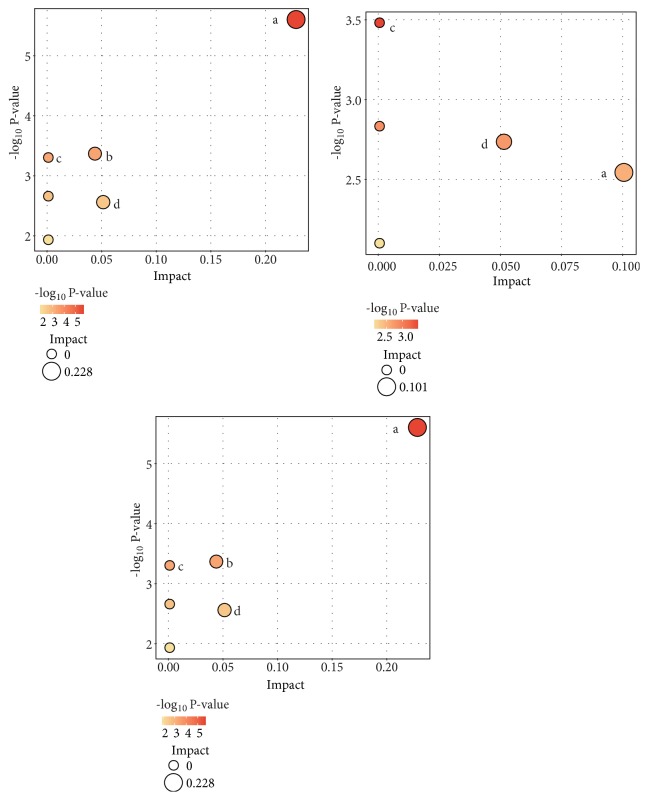
Summary of pathway analysis by lipidomics (A: NSCLC versus HC; B: NSCLC versus LBD; C: LBD versus HC; a: glycerophospholipid metabolism; b: glycosylphosphatidylinositol- (GPI-) anchor biosynthesis; c: linoleic acid metabolism; d: glycerolipid metabolism;). NSCLC, non-small cell lung cancer; LBD, lung benign disease; HC, healthy controls.

The lipid metabolite features with variable importance in projection value (VIP) > 1.0, fold change (FC) >1.5, and P value < 0.05 were as the potential different lipid metabolites. As summarized in Tables 3–5. There were 60 specific lipid metabolites that can distinguish NSCLC from HC, 8 for NSCLC from LBD, and 44 for LBD from HC. PCs and PEs were significantly upregulated in serum of early-stage NSCLC compared to HC and LBD, which should be further externally validated by a targeted lipidomic analysis. The pathways that matched based on Kyoto Encyclopedia of Genes and Genomes (KEGG) database included glycerophospholipid metabolism, glycosylphosphatidylinositol- (GPI-) anchor biosynthesis, linoleic acid metabolism, alpha-linolenic acid metabolism, and glycerolipid metabolism (Figure 4).  Table 6 listed the detailed results of the pathway analysis. Glycerophospholipid (GPL) pathway emerged at the top of these significantly altered lipid metabolic pathways.

### 3.3. Targeted Metabolomics Analysis

**Figure 5:**
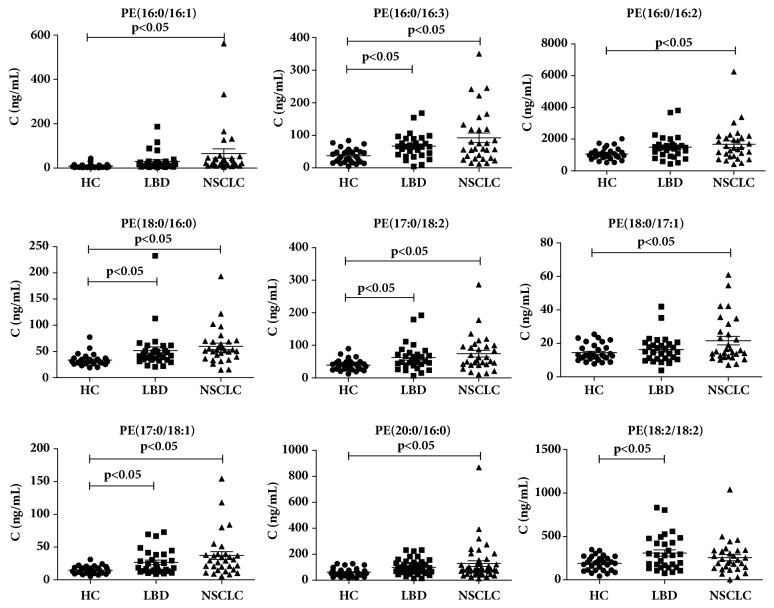
Bar graph of concentrations of discrepant biomarkers among early-stage NSCLC, lung benign disease (LBD), and healthy controls (HC) groups. The black horizontal lines are median values. P values are determined by Fisher's least significant test.

**Figure 6:**
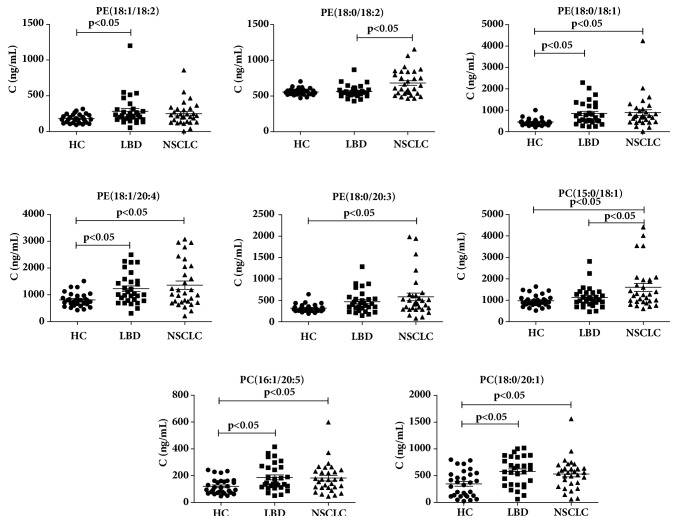
Bar graph of concentrations of discrepant biomarkers among early-stage NSCLC, lung benign disease (LBD), and healthy controls (HC) groups. The black horizontal lines are median values. P values are determined by Fisher's least significant test.

We analyzed the change in the concentrations of 85 PCs and 53 PEs in the early-stage NSCLC, LBD, and HC groups. The levels of PCs and PEs were compared among the three groups using ANOVA with LSD test. The fold changes of the average of the concentrations of PCs and PEs were also calculated among them. As shown in Table 7, 11 PEs and 3 PCs were selected as biomarkers for distinguishing early-stage NSCLC and HC according to the p<0.05 and fold change >1.5. 8 PEs and 2 PCs were selected as biomarkers for distinguishing LBD and HC according to the p<0.05 and fold change >1.5. One PE and 1 PC were selected as biomarkers for distinguishing early-stage NSCLC and LBD according to the p<0.05 and fold change >1.0. The concentration distributions of these selected PCs and PEs were shown in Figures 5 and 6. As shown in Figures 5 and 6, significant increases in the levels of PCs and PEs in early-stage NSCLC were observed compared with LBD and HC, whereas the concentrations of these PCs and PEs in LBD were significantly increased relative to HC. The levels of PE(16:0/16:1), PE(16:0/18:3), PE(16:0/18:2), PE(18:0/16:0), PE(17:0/18:2), PE(18:0/17:1), PE(17:0/18:1), PE(20:5/16:0), PE(18:0/18:1), PE(18:1/20:4), PE(18:0/20:3), PC(15:0/18:1), PC(16:1/20:5), and PC(18:0/20:1) in early-stage NSCLC were significantly increased compared with HC (p<0.05). The levels of PE(16:0/18:3), PE(18:0/16:0), PE(17:0/18:2), PE(17:0/18:1), PE(18:2/18:2), PE(18:1/18:2), PE(18:0/18:1), PE(18:1/20:4), PC(16:1/20:5), and PC(18:0/20:1) in LBD were significantly increased compared with HC (p<0.05). The levels of PE(18:0/18:2) and PC(15:0/18:1) in early-stage NSCLC were significantly increased compared with LBD (p<0.05).

**Figure 7:**
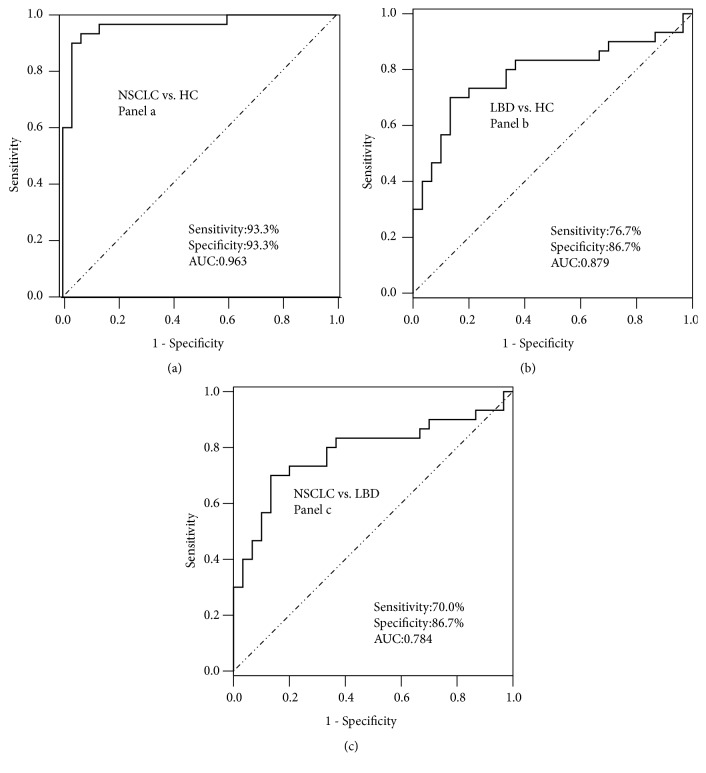
Representative ROC curves between early-stage NSCLC, LBD, and HC. (a) Panel a for differentiating early-stage NSCLC from HC. (b) Panel b for differentiating LBD from HC. (c) Panel c for differentiating early-stage NSCLC from LBD. NSCLC, non-small cell lung cancer; LBD, lung benign disease; HC, healthy controls.

To estimate the diagnostic value of the targeted PCs and PEs, ROC analysis was further performed. The sensitivity, specificity, and area under the curve (AUC) of each lipid metabolite and the combination of PCs and PEs were presented in Table 7. It was found that single PC and PE did not have good diagnostic performance in distinguishing NSCLC from LBD or HC. However, as showed in Figure 7, the combination of 14 PCs and PEs (Panel a) had the best diagnostic performance for distinguishing early-stage NSCLC from HC (AUC=0.963). The combination of 10 PCs and PEs (Panel b) had the best diagnostic performance for distinguishing LBD from HC (AUC=0.879). The combination of 2 PCs and PEs (Panel c) had the best diagnostic performance for distinguishing early-stage NSCLC from LBD (AUC=0.784).

## 4. Discussion

NSCLC is the most frequently diagnosed cancer with high mortality, partly ascribed to late diagnosis and poor prognosis. Many of the commonly used serum tumor biomarkers are limited to late-stage disease and have low sensitivity and specificity [32, 33]. Currently, there are a handful of validated small molecular biomarkers for NSCLC that can be used to avoid the necessity of tumor biopsies for classifying NSCLC. But a new diagnostic technique with high accuracy for the diagnosis of NSCLC, particularly for distinguishing early cancer from benign lesions, is still needed in clinical practice.

Lipids were hydrophobic or amphipathic small molecules that originate entirely or in part by carbanion-based condensations of thioesters and/or by carbocation-based condensations of isoprene units [34]. Many studies have reported that dyslipidemia, as a major component of metabolic syndrome, played an important role in the carcinogenesis of various cancers, including breast cancer [35], prostate cancer [36], and ovarian cancer [37]. For NSCLC, it has been well documented that lipidomics have shown potential for cancer diagnosis [27, 38, 39]. In our study, we identified PCs and PEs showing significant differences of serum concentration among HC, early-stage NSCLC, and LBD patients. GPL metabolism was the top altered pathway in the NSCLC samples. The serum concentrations of PCs and PEs were shown to increase in the NSCLC patients, while the others decreased. These results might be caused by the regulation mechanisms of cellular metabolism.

Phospholipids, one of the major components of cell membranes, participate in various biological functions, and their levels are altered in various human cancers [40, 41]. PCs were known as the most abundant bilayer-forming phospholipids found in eukaryotic membranes and can contribute to proliferative growth in cancer cells [42, 43]. Abnormal PC metabolism has been reported in cancer cells. Increased PCs levels have been reported in lung cancer, colorectal cancer, gastric cancer, pancreatic cancer, and so on and thus might be interpreted as a requirement for the high rate of cancer cell proliferation [44]. Additionally, increased levels of PCs may be correlated with the overexpression of choline kinase in various cancers [45]. In our study, the levels of PC(15:0/18:1), PC(16:1/20:5), and PC(18:0/20:1) in early-stage NSCLC patients were significantly increased compared with LBD patients and HC.

PE was the second most abundant phospholipid in mammalian cells. It had quite remarkable activities and had roles in the regulation of cell proliferation, metabolism, organelle function, endocytosis, autophagy, stress responses, apoptosis, and aging. PE was also a target of potent anticancer natural products [46]. In our study, the levels of PEs in early-stage NSCLC patients were significantly increased compared with LBD and HC. Consistent with our finding, Fahrmann et al. also found that PEs tended to be elevated in serum from lung cancer patients compared to those with benign nodules [47]. Aberrant PE metabolism was also detected in other cancers, such as hepatocellular carcinoma, colorectal cancer, and breast tumor [48]. Huang et al. previously illustrated that A549 lung adenocarcinoma cells increase secretion of PE binding protein (PEBP), which was overexpressed in lung cancer and had been shown to modulate development, invasion, and metastatic potential of tumors [49]. Thus, we speculated that the elevation in PEs may, in part, act as agonists of PEBP-mediated signaling transduction. PE was found to consistently increase in tumors similar to PC. In our study, we found that single PC and PE did not have good diagnostic performance in distinguishing NSCLC from LBD or HC. Panels of PCs and PEs exhibited good performance in differentiating NSCLC, LBD patients, and HC, which should be further validated by a larger sample sizes.

## 5. Conclusions

We observed a significantly altered lipid metabolic profile in early-stage NSCLC using UHPLC-Q-TOF/MS-based nontargeted lipidomic analysis and identified panels of PCs and PEs to distinguish NSCLC, LBD patients, and HC. The identified PCs and PEs were further externally validated by a targeted lipidomic analysis. ROC analysis revealed that a panel of 14 PCs and PEs exhibited good performance in differentiating HC and early-stage NSCLC patients. A panel of 10 PCs and PEs exhibited good performance in differentiating HC and LBD patients. A panel of 2 PCs and PEs exhibited good performance in differentiating early-stage NSCLC and LBD patients. Our study has thus highlighted the power of using comprehensive lipidomic approaches to identify biomarkers and underlying mechanisms in NSCLC.

## Figures and Tables

**Table 1:** The clinical characteristics of early-stage NSCLC, HC, and LBD enrolled in this study.

Characteristics	Non-targeted lipidomics			Targeted lipidomics		
	NSCLC	LBD	HC	NSCLC	LBD	HC
Sample size	66	40	40	30	30	30
Age range (years)	61.5±8.2	59.2±10.0	54.0±7.5	62.1±6.7	53.9±11.2	51.7±7.1
Gender						
male	40	21	24	21	18	19
female	26	19	16	9	12	11
Pathological type						
ADC	35	-* *-	-* *-	15	-* *-	-* *-
SqCC	31	-* *-	-* *-	15	-* *-	-* *-
TNM Stages*∗*						
Stage I	38	-* *-	-* *-	15	-* *-	-* *-
Stage II	28	-* *-	-* *-	15	-* *-	-* *-

*∗*Union for International Cancer Control (UICC) TNM Classification of Lung Cancer (8th ed., 2017).

NSCLC, non-small cell lung cancer; LBD, lung benign disease; HC, healthy controls; ADC, lung adenocarcinoma; SqCC, lung squamous cell carcinoma.

**Table 2:** The parameters of the OPLS-DA models.

Group	R^2^X	R^2^Y	Q^2^
NSCLC vs HC	0.296	0.803	0.739
NSCLC vs LBD	0.255	0.706	0.481
LBD vs HC	0.263	0.883	0.762

NSCLC, non-small cell lung cancer; LBD, lung benign disease; HC, healthy controls.

**Table 3:** Statistical analysis of differential lipids to distinguish early-stage NSCLC from HC group.

Peak	Lipid	Polarity	VIP value	Fold Change	p value	Peak	Lipid	Polarity	VIP value	Fold Change	p value	Peak	Lipid	Polarity	VIP value	Fold Change	p value
1	PC(P-18:0/15:1)	POS	1.22	10.41	<0.001	21	TG(14:0/16:1/18:1)	POS	1.37	1.73	<0.001	41	TG(17:0/17:2/19:0)	POS	1.29	1.54	<0.001
2	TG(14:0/16:0/16:1)	POS	1.21	2.39	0.011	22	PE(24:4/12:0)	POS	1.88	1.72	<0.001	42	PC(18:3/22:6)	POS	1.04	1.54	<0.001
3	DG(18:1/18:1)	POS	2.05	2.28	<0.001	23	TG(16:1/18:3/18:3)	POS	1.06	1.70	<0.001	43	TG(16:0/18:0/18:0)	POS	1.48	1.53	<0.001
4	TG(14:0/16:0/18:1)	POS	1.64	2.27	<0.001	24	PC(P-20:0/19:0)	POS	1.60	1.68	<0.001	44	TG(16:0/16:1/18:3)	POS	1.28	1.53	<0.001
5	CE(18:4)	POS	1.46	2.03	<0.001	25	PC(18:3/26:2)	POS	1.37	1.67	<0.001	45	TG(17:0/18:1/20:4)	POS	1.15	1.53	0.002
6	DG(16:0/16:0)	POS	1.65	1.99	<0.001	26	PC(18:1/24:4)	POS	1.78	1.67	<0.001	46	TG(16:1/16:1/17:0)	POS	1.21	1.53	0.002
7	PC(16:0/24:4)	POS	2.03	1.96	<0.001	27	TG(16:0/16:0/18:1)	POS	1.75	1.67	<0.001	47	PC(9:0/26:1)	POS	1.77	1.50	<0.001
8	PE(22:4/14:1)	POS	1.34	1.96	<0.001	28	TG(14:1/16:1/18:1)	POS	1.32	1.64	0.003	48	PC(18:0/22:4)	NEG	1.84	1.83	<0.001
9	TG(14:0/16:0/16:0)	POS	1.22	1.96	<0.001	29	PC(14:1/26:2)	POS	2.00	1.64	<0.001	49	PC(20:5/20:4)	NEG	1.72	1.82	<0.001
10	PI(12:0/24:4)	POS	1.85	1.93	<0.001	30	PC(4:0/26:1)	POS	1.33	1.61	0.014	50	PC(18:0/22:5)	NEG	1.88	1.80	<0.001
11	DG(18:1/18:0)	POS	1.77	1.87	<0.001	31	PC(6:0/26:0)	POS	1.70	1.60	<0.001	51	PC(P-18:0/4:0)	NEG	1.20	1.72	<0.001
12	TG(17:0/18:0/18:1)	POS	1.46	1.86	<0.001	32	PC(12:0/26:2)	POS	1.80	1.60	<0.001	52	PC(18:0/18:0)	NEG	2.23	1.72	<0.001
13	TG(14:1/16:1/16:1)	POS	1.22	1.83	0.011	33	Cer(d18:1/24:1)	POS	1.94	1.59	<0.001	53	PC(18:0/20:3)	NEG	1.89	1.67	<0.001
14	TG(16:0/17:0/18:1)	POS	1.51	1.83	<0.001	34	TG(19:0/19:0/21:0)	POS	1.23	1.58	<0.001	54	PC(P-20:0/24:4)	NEG	1.71	1.64	<0.001
15	TG(18:0/18:0/18:0)	POS	1.34	1.83	<0.001	35	PE(22:2/12:0)	POS	1.71	1.58	<0.001	55	PC(16:0/20:5)	NEG	1.48	1.63	<0.001
16	TG(17:0/17:0/17:1)	POS	1.02	1.81	0.019	36	PE(24:4/14:1)	POS	1.51	1.58	<0.001	56	PC(22:1/18:2)	NEG	1.41	1.58	<0.001
17	PC(6:0/26:1)	POS	1.71	1.76	<0.001	37	PC(18:3/24:4)	POS	1.86	1.56	<0.001	57	PC(18:0/20:2)	NEG	1.83	1.57	<0.001
18	SM(d18:0/26:1)	POS	1.41	1.75	<0.001	38	SM(d16:0/26:0)	POS	1.48	1.56	<0.001	58	PC(16:0/16:0)	NEG	1.78	1.54	<0.001
19	PC(10:0/26:0)	POS	2.06	1.74	<0.001	39	TG(16:0/16:0/16:0)	POS	1.12	1.55	<0.001	59	PC(17:0/18:1)	NEG	1.82	1.50	<0.001
20	TG(16:1/18:2/18:3)	POS	1.30	1.73	<0.001	40	PC(18:2/24:4)	POS	1.63	1.54	<0.001	60	PC(18:0/20:4)	NEG	1.84	1.50	<0.001

NSCLC, non-small cell lung cancer; HC, healthy controls.

**Table 4:** Statistical analysis of differential lipids to distinguish early-stage NSCLC from LBD group.

Peak	Lipid	Polarity	p value	VIP value	Fold Change
1	PC(22:5/10:0)	POS	<0.001	1.67	1.73
2	PE(P-18:0/18:2)	POS	<0.001	2.71	1.68
3	PC(2:0/17:1)	POS	0.003	1.63	1.58
4	PC(2:0/17:2)	POS	0.003	1.54	1.56
5	PC(3:0/18:4)	POS	0.006	1.70	1.56
6	TG(12:0/18:1/16:1)	POS	<0.001	2.59	1.55
7	PI(16:0/16:1)	NEG	0.033	1.22	1.57
8	PC(18:4/3:0)	NEG	0.008	1.66	1.50

NSCLC, non-small cell lung cancer; LBD, lung benign disease.

**Table 5:** Statistical analysis of differential lipids to distinguish LBD from HC group.

Peak	Lipid	Polarity	VIP value	Fold Change	p value	Peak	Lipid	Polarity	VIP value	Fold Change	p value
1	PC(P-18:0/15:1)	POS	2.19	13.46	<0.001	23	PC(16:0/20:5)	NEG	1.40	1.73	<0.001
2	PC(16:0/24:4)	POS	1.35	1.65	<0.001	24	PE(16:0/22:6)	NEG	2.04	1.71	<0.001
3	PC(18:2/24:4)	POS	1.24	1.61	<0.001	25	PE(18:0/0:0)	NEG	1.70	1.71	<0.001
4	PC(18:1/24:4)	POS	1.08	1.53	<0.001	26	PE(18:0/20:4)	NEG	1.74	1.70	<0.001
5	PC(18:3/26:2)	POS	1.15	1.52	<0.001	27	PE(17:0/18:1)	NEG	1.97	1.68	<0.001
6	PI(18:1/18:2)	NEG	1.91	2.34	0.006	28	PI(17:0/20:4)	NEG	1.70	1.67	<0.001
7	PE(20:1/16:0)	NEG	1.89	2.05	<0.001	29	PI(18:0/22:5)	NEG	1.93	1.65	<0.001
8	PI(16:0/16:1)	NEG	1.66	2.05	0.003	30	PC(20:5/20:4)	NEG	1.75	1.64	<0.001
9	PE(18:0/22:5)	NEG	2.20	1.98	<0.001	31	PC(20:5/0:0)	NEG	1.41	1.63	<0.001
10	PE(16:0/20:5)	NEG	1.82	1.92	<0.001	32	PC(18:0/22:5)	NEG	2.04	1.63	<0.001
11	PI(18:0/22:4)	NEG	2.00	1.91	<0.001	33	PE(22:5/18:0)	NEG	1.60	1.62	<0.001
12	PE(18:1/20:4)	NEG	1.69	1.88	<0.001	34	PG(24:0/18:0)	NEG	2.03	1.60	<0.001
13	PE(20:1/24:1)	NEG	1.98	1.88	<0.001	35	PI(16:0/22:6)	NEG	1.53	1.59	<0.001
14	PI(16:0/22:5)	NEG	1.66	1.87	<0.001	36	PC(18:0/22:4)	NEG	1.70	1.59	<0.001
15	PI(16:0/20:5)	NEG	1.91	1.85	<0.001	37	PE(18:0/20:3)	NEG	1.85	1.58	<0.001
16	PE(16:0/18:3)	NEG	1.93	1.83	<0.001	38	PI(18:0/20:3)	NEG	1.59	1.56	<0.001
17	PE(16:0/20:4)	NEG	1.47	1.80	<0.001	39	PE(18:3/0:0)	NEG	1.62	1.54	<0.001
18	PE(22:6/16:0)	NEG	1.53	1.78	<0.001	40	PE(20:3/0:0)	NEG	1.57	1.52	<0.001
19	PC(P-18:0/4:0)	NEG	1.48	1.78	<0.001	41	PC(16:1/0:0)	NEG	1.82	1.52	<0.001
20	PE(18:1/22:6)	NEG	1.62	1.76	<0.001	42	PC(18:0/20:3)	NEG	1.58	1.51	<0.001
21	PE(16:0/0:0)	NEG	1.76	1.75	<0.001	43	PC(20:1/22:6)	NEG	1.58	1.50	<0.001
22	PE(18:0/18:2)	NEG	1.52	1.75	<0.001	44	PE(20:5/0:0)	NEG	1.48	1.50	<0.001

LBD, lung benign disease; HC, healthy controls.

**Table 6:** The detailed results of the pathway analysis by lipidomics.

Groups		Total	Hits	Raw *p*	-log(p)	FDR	Impact
NSCLC vs HC	Glycerophospholipid metabolism	39	3	0.00002	11.070	0.001	0.329
	Glycosylphosphatidylinositol(GPI)-anchor biosynthesis	14	1	0.023	3.769	0.659	0.044
	Linoleic acid metabolism	15	1	0.025	3.701	0.659	0.001
	alpha-Linolenic acid metabolism	29	1	0.047	3.050	0.835	0.001
	Glycerolipid metabolism	32	1	0.052	2.954	0.835	0.012

NSCLC vs LBD	Glycerophospholipid metabolism	39	2	0.001	7.182	0.061	0.228
	Glycosylphosphatidylinositol(GPI)-anchor biosynthesis	14	1	0.017	4.054	0.496	0.044
	Linoleic acid metabolism	15	1	0.019	3.985	0.496	0.001
	alpha-Linolenic acid metabolism	29	1	0.036	3.332	0.715	0.001

LBD vs HC	Glycerophospholipid metabolism	39	3	0.00002	11.070	0.001	0.329
	Glycosylphosphatidylinositol(GPI)-anchor biosynthesis	14	1	0.023	3.769	0.659	0.044
	Linoleic acid metabolism	15	1	0.025	3.701	0.659	0.001
	alpha-Linolenic acid metabolism	29	1	0.047	3.050	0.835	0.001
	Glycerolipid metabolism	32	1	0.052	2.954	0.835	0.012

NSCLC, non-small cell lung cancer; LBD, lung benign disease; HC, healthy controls.

**Table 7:** The detection of PCs and PEs as potential biomarkers for distinguishing early-stage NSCLC, LBD, and HC.

Lipid biomarker	NSCLC vs. HC			LBD vs. HC			NSCLC vs. LBD		
	AUC (95%CI)	Sensitivity (%)	Specificity (%)	AUC (95%CI)	Sensitivity (%)	Specificity (%)	AUC (95%CI)	Sensitivity (%)	Specificity (%)
PE(16:0/16:1)	0.900(0.822-0.978)	80.0	93.3	-* *-			-* *-	-* *-	-* *-
PE(16:0/18:3)	0.741(0.614-0.868)	66.7	80.0	0.763(0.640-0.887)	70.0	80.0	-* *-	-* *-	-* *-
PE(16:0/18:2)	0.698(0.559-0.837)	60.0	80.0	-* *-			-* *-	-* *-	-* *-
PE(18:0/16:0)	0.820(0.703-0.937)	70.0	93.3	0.760(0.633-0.887)	66.7	83.3	-* *-	-* *-	-* *-
PE(17:0/18:2)	0.731(0.599-0.863)	56.7	83.3	0.703(0.568-0.839)	70.0	70.0	-* *-	-* *-	-* *-
PE(18:0/17:1)	0.657(0.517-0.796)	66.7	66.7	-* *-			-* *-	-* *-	-* *-
PE(17:0/18:1)	0.782(0.659-0.906)	73.3	80.0	0.709(0.578-0.840)	46.7	93.3	-* *-	-* *-	-* *-
PE(20:5/16:0)	0.759(0.631-0.887)	76.7	76.7	-* *-			-* *-	-* *-	-* *-
PE(18:2/18:2)	-* *-			0.663(0.523-0.804)	53.3	86.7	-* *-	-* *-	-* *-
PE(18:1/18:2)	-* *-			0.686(0.551-0.820)	63.3	70.0	-* *-	-* *-	-* *-
PE(18:0/18:2)	-* *-			-* *-			0.686(0.547-0.824)	43.3	96.7
PE(18:0/18:1)	0.783(0.658-0.908)	73.3	83.3	0.781(0.655-0.907)	80.0	80.0	-* *-	-* *-	-* *-
PE(18:1/20:4)	0.691(0.554-0.828)	60.0	76.7	0.734(0.606-0.862)	70.0	73.3	-* *-	-* *-	-* *-
PE(18:0/20:3)	0.733(0.600-0.867)	50.0	96.7	-* *-			-* *-	-* *-	-* *-
PC(15:0/18:1)	0.717(0.586-0.848)	60.0	80.0	-* *-			0.643(0.503-0.784)	36.7	93.3
PC(16:1/20:5)	0.687(0.550-0.823)	53.3	86.7	0.709(0.577-0.841)	86.7	50.0	-* *-	-* *-	-* *-
PC(18:0/20:1)	0.691(0.556-0.826)	66.7	70.0	0.739(0.615-0.863)	63.3	76.7	-* *-	-* *-	-* *-
Panel a	0.963(0.916-1.000)	93.3	93.3	-* *-			-* *-	-* *-	-* *-
Panel b	-* *-			0.879(0.789-0.969)	76.7	86.7	-* *-	-* *-	-* *-
Panel c	-* *-			-* *-			0.784(0.662-0.906)	70.0	86.7

NSCLC, non-small cell lung cancer; LBD, lung benign disease; HC, healthy controls.

Panel a: PE(16:0/16:1), PE(16:0/18:3), PE(16:0/18:2), PE(18:0/16:0), PE(17:0/18:2), PE(18:0/17:1), PE(17:0/18:1), PE(20:5/16:0), PE(18:0/18:1), PE(18:1/20:4), PE(18:0/20:3), PC(15:0/18:1, PC(16:1/20:5), and PC(18:0/20:1); Panel b: PE(16:0/18:3), PE(18:0/16:0), PE(17:0/18:2), PE(17:0/18:1), PE(18:2/18:2), PE(18:1/18:2), PE(18:0/18:1), PE(18:1/20:4), PC(16:1/20:5), and PC(18:0/20:1); Panel c: PE(18:0/18:2) and PC(15:0/18:1).

## Data Availability

The data used to support the findings of this study are included within the article and its supplementary information files.
